# Supplementary material for: Structure of the TnsB transposase-DNA complex of type V-K CRISPR-associated transposon
Source: Nat Commun. 2022 Oct 2;13:5792. doi: 10.1038/s41467-022-33504-5 (PMC9527255; doi:10.1038/s41467-022-33504-5)
Supplement: Supplementary file 1 — Supplementary Information [file 41467_2022_33504_MOESM1_ESM.pdf]

Supplementary Materials

Structure of the TnsB transposase-DNA complex of type V-K CRISPR-associated transposon

Francisco Tenjo-Castaño[1], Nicholas Sofos[1], Blanca López-Méndez[2], Luisa S. Stutzke[1], Anders Fuglsang[1], Stefano Stella[1‡] and Guillermo Montoya[1*]

[1]Structural Molecular Biology Group, Novo Nordisk Foundation Centre for Protein Research, Faculty of Health and Medical Sciences University of Copenhagen; Copenhagen, 2200, Denmark.

[2]Protein Purification and Characterisation Facility, Novo Nordisk Foundation Centre for Protein Research, Faculty of Health and Medical Sciences University of Copenhagen; Copenhagen, 2200, Denmark.

INDEX

Supplementary Figures 1-8
Supplementary Movie Legend
Supplementary Tables 1-2

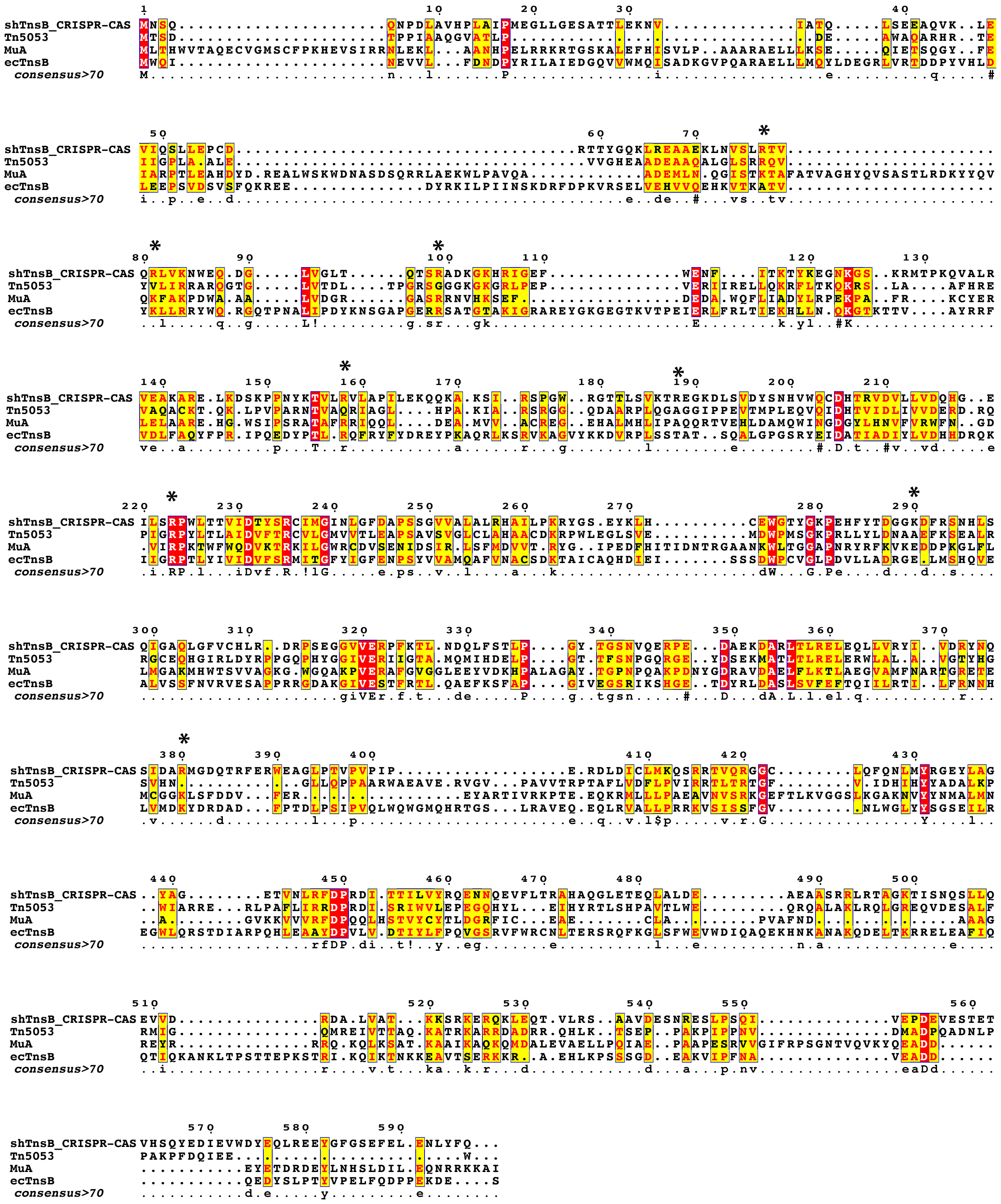

Supplementary Figure 1.- Sequence alignment of representative members of the DDE transposase family. The amino acid sequences of EcTnsB, shTnsB, MuA and Tn5053 were aligned by Clustal Omega (http://www.ebi.ac.uk/Tools/msa/clustalo). The figure was prepared with ESPript (http://espript.ibcp.fr). Residue numbers are labelled according to shTnsB sequence. Similar residues are shown in red and identical residues in white over red background. The different structural domains of shTnsB and their amino acid composition are depicted as boxes above the sequences and labelled with the same names and colour code as in Fig. 1a. The mutations performed in key residues commented through the text are marked with an asterisk *.

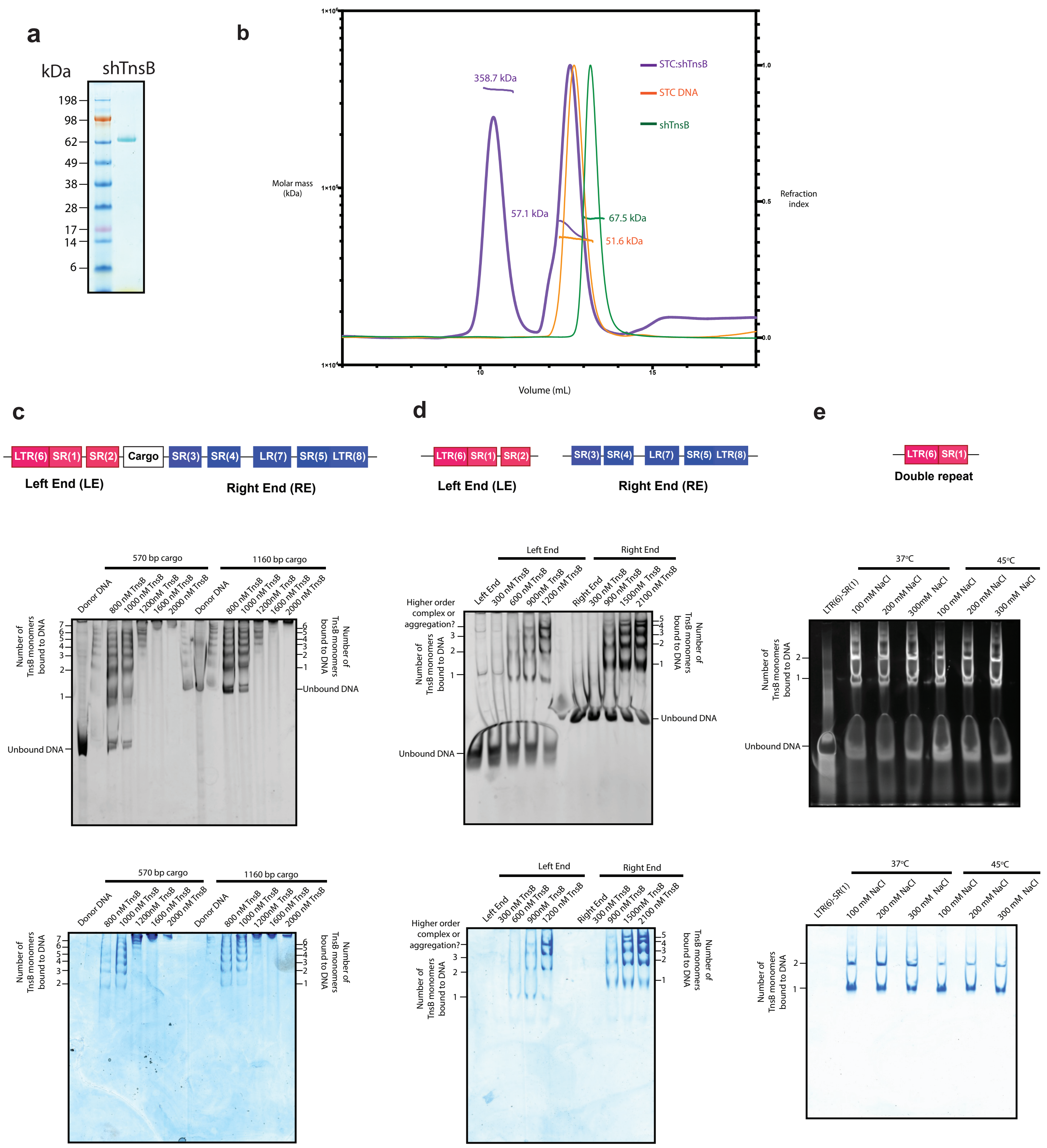

f

Left end

```
LTR(6)      TGTACAGTGACAAATTATCTGTCGT
SR(1)            GTGACAGATTAA-TGTCAT
SR(2)            ATGACAAATTAAATGTCA
```

Right end

```
SR(3)            ATGACAAATTGACTGTCG
SR(4)            GTGACAAATTAGCTGTCG
LR(7)       TTTGCAGTGACAAAATAGATGTCGT
SR(5)            GTGACAAATTAT-TGTCAT
LTR(8)      TGTACAGTGACTAATTATATGTCGT
```

Supplementary Figure 2.- Purification and characterisation of shTnsB and the Strand Transfer Complex. a) SDS-PAGE showing the purified shTnsB **b)** SEC-MALS of purified TnsB (green), STC DNA (orange), and shTnsB:STC (purple). The results agree with the theoretical molecular weight of a TnsB

monomer (68 kDa) and the separate target bound RE and LE DNA (52.6 kDa and 52 kDa). As for the complex, the observed molecular weight (358.7 kDa) corresponds to a complex containing the target bound RE and LE and a TnsB tetramer, which has a theoretical molecular weight of 376.6 kDa. **c) Electromobility shift assay (EMSA) from** complexes containing donor DNA with two different cargos. Donor DNA concentration was 100 nM in all samples. TnsB concentration in each sample is indicated in the figure. **d) EMSA from** complexes containing only RE or LE. Donor DNA concentration was 300 nM in all samples. TnsB concentration in each sample is indicated in the figure. **e) EMSA from** complex containing only LTR(6) and SR(1) tested at different temperatures and NaCl concentrations. DNA concentration was 2 μM and TnsB concentration was 4 μM. All EMSA experiments were performed twice independently producing similar results. **f)** Alignment of the different long terminal repeats (LTR), short repeats (SR) and long repeat (LR) in the RE and LE.

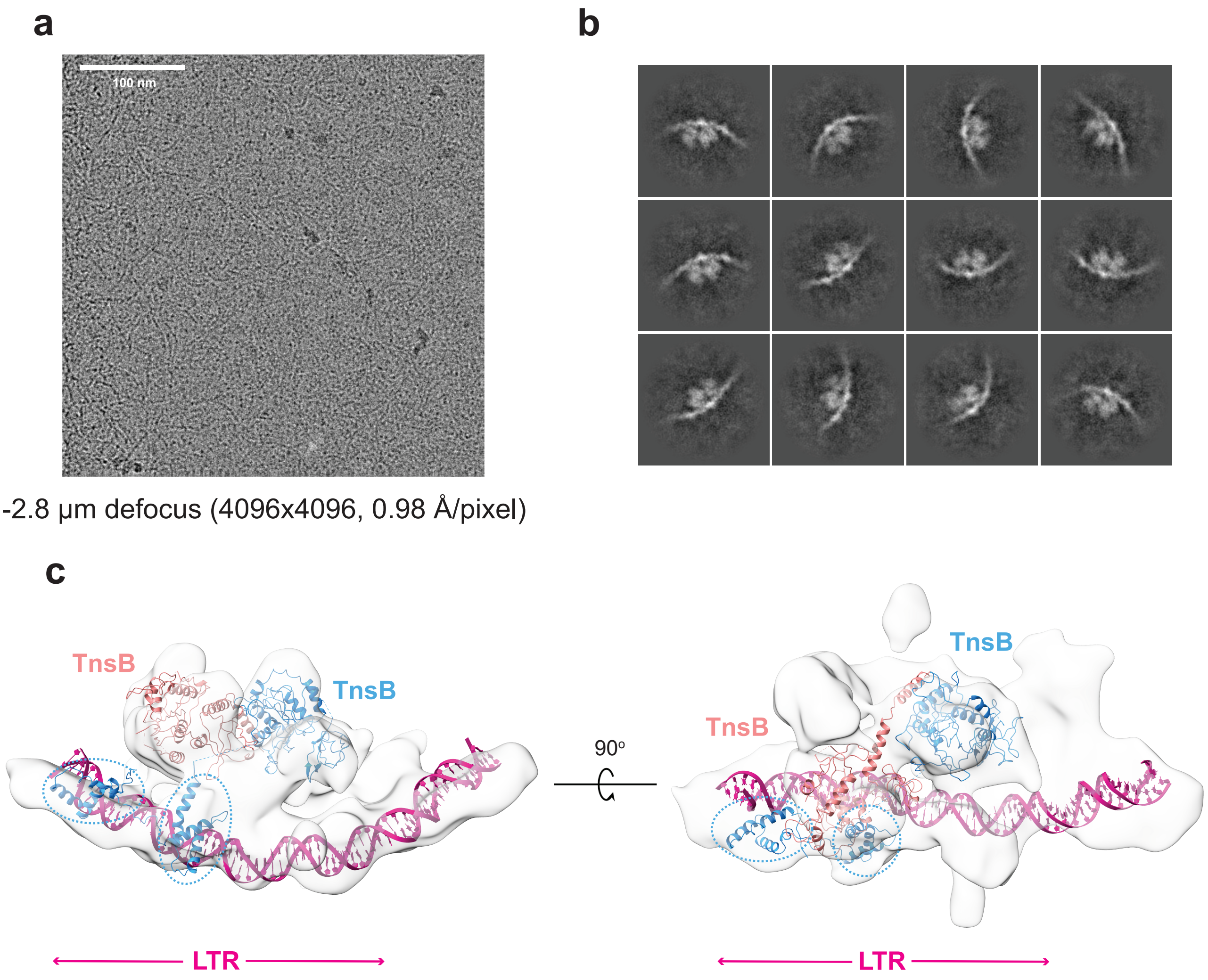

Supplementary Figure 3.- Low resolution reconstruction of the shTnsB bound to RE/LE in the precatalytic state. a) Representative cryo-EM micrograph (out of a total of 738 micrographs) of this protein-DNA complex in vitreous ice on an UltrAuFoil 1.2/1.3 grid at -2.8 µm defocus. **b)** Reference-free 2D class averages selected for *ab inito* reconstruction displaying a bent DNA molecule bound to two protomers of shTnsB. **c)** 3D reconstruction based on selected 2D classes. Two protomers of shTnsB were fitted into the density based on the STC structure and following a similar pattern to the precatalytic structure of ecTnsB [19]. NTD1 and NTD2 are shown in dashed circles indicating where the LTR would be located.

Oligonucleotide	Sequence (5'⟶3')
RE_Target - Chain I (TS)	ATAAGGATTTTACTGATGACAATAATTTGTCA CAACGACATATAATTAGTCACTGTACACGTAG AGACGTAGCAATGCT
RE_polyA - Chain J (NTS)	AAAAAAAAAAAAAAAATGTACAGTGACTAATTA TATGTCGTTGTGACAAATTATTGTCATCAGTA AAATCCTTAT
Target_1 - Chain K	AGCATTGCTACGTCT
LE_Target - Chain L (TS)	AATTAAATAGTCACAATGACATTAATCTGTCA CCGACGACAGATAATTTGTCACTGTACACTAC GCCTTTTGTGGAGATG
LE_polyA - Chain M (NTS)	AAAAAAAAAAAAAAAATGTACAGTGACAAATTA TCTGTCGTCGGTGACAGATTAATGTCATTGTG ACTATTTAATT
Target_2 - Chain N	CATCTCCACAAAAGG

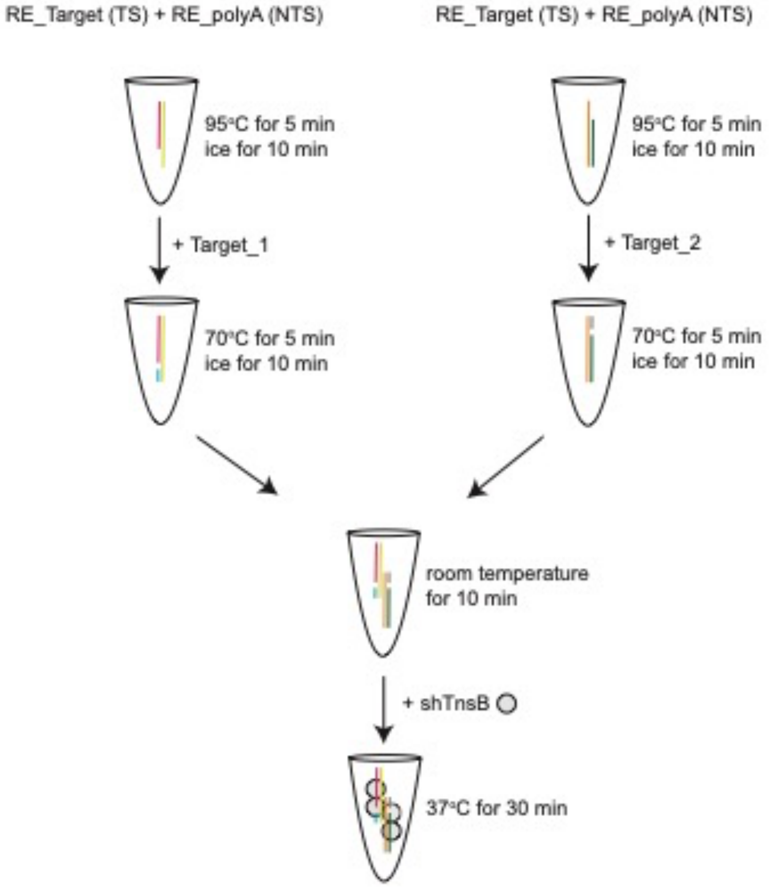

Supplementary Figure 4.- Overview of the shTnsB-STC DNA reconstitution process. Sequential mixing of the ssDNA corresponding to transferred strands (TS) and non-transferred strands (NTS) from RE and LE was performed in three steps before adding shTnsB. The upper table shows the sequence of each ssDNA and their specific name following the nomenclature in Supplementary Table 1. Colors follow the same code used in Figure 1 and Supplementary Figure 7.

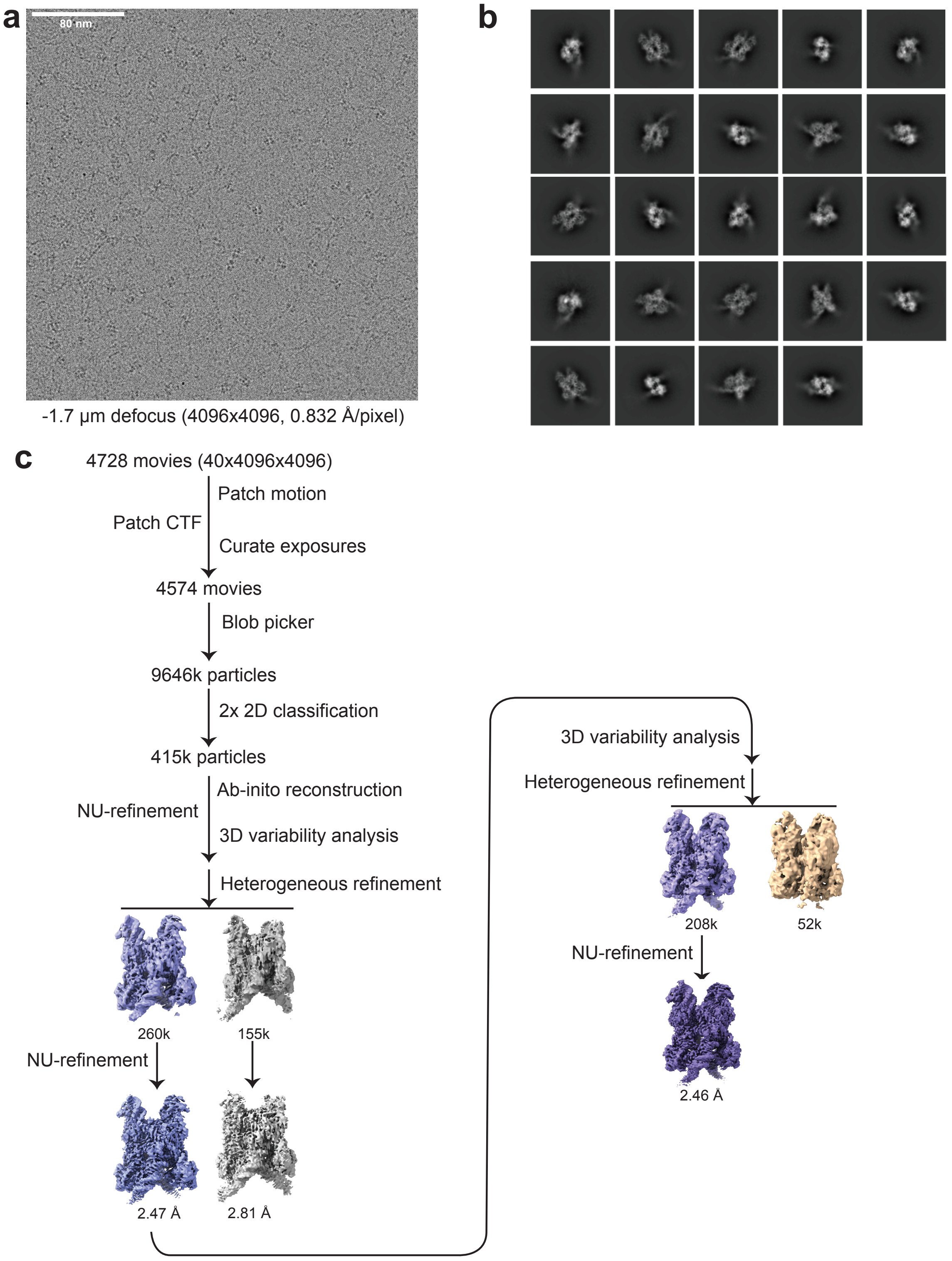

Supplementary Figure 5.- Single Particle Cryo-EM Analysis of the TnsB-STC. (a) Representative cryo-EM micrograph (out of a total of 4728 micrographs) of the TnsB-STC in vitreous ice on an UltrAuFoil 1.2/1.3 grid at -1.7 µm defocus. **(b)** Reference-free 2D class averages selected for *ab inito* reconstruction. **(c)** Overview of the cryo-EM data processing workflow as performed in cryoSPARC.

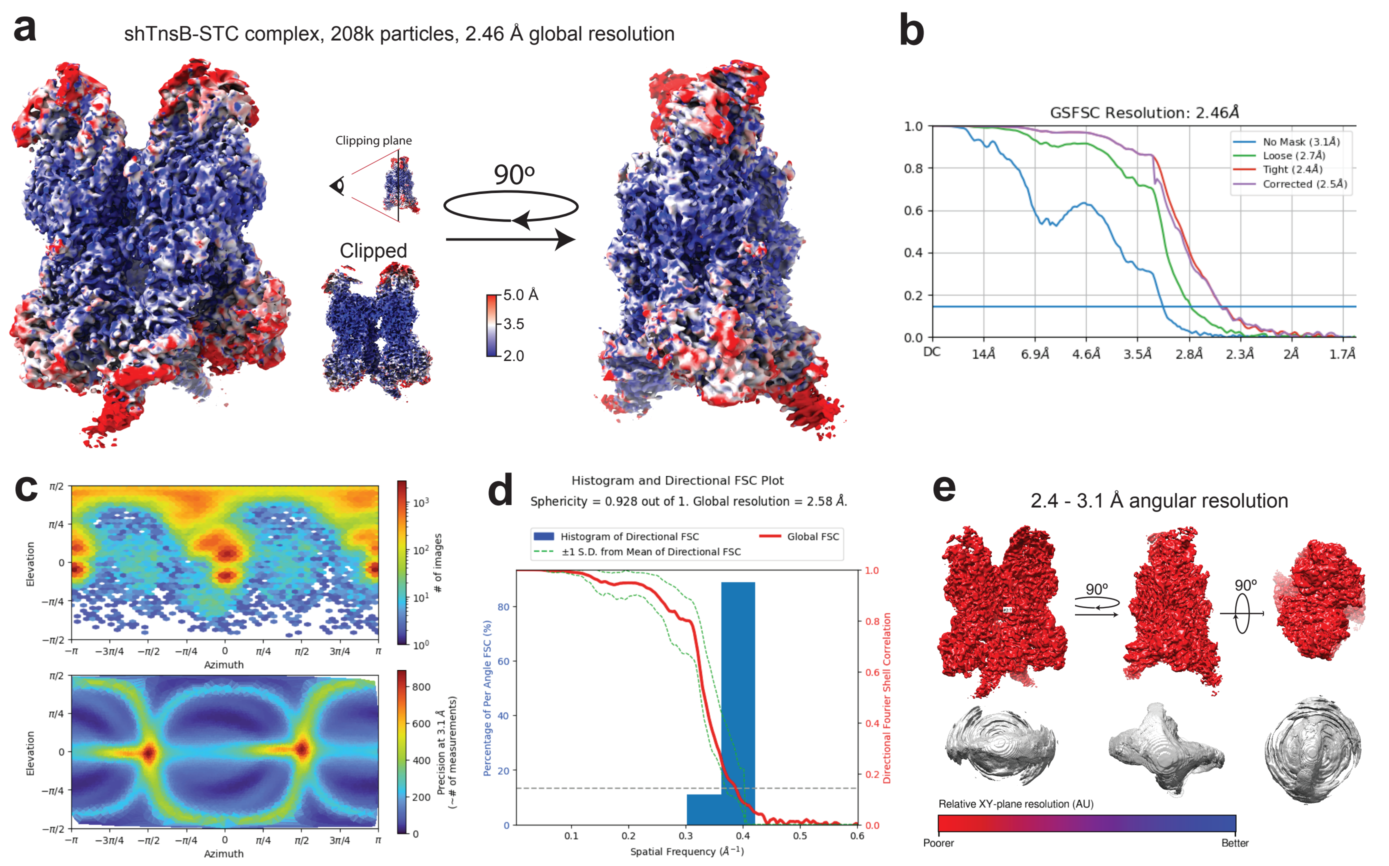

Supplementary Figure 6.- Resolution Assessment and Validation of the TnsB-STC Cryo-EM map (a) Local resolution maps in two orientation related by a 90° rotation around the y-axis. The inset shows the high resolution obtained in the central cut through the map, the clipping plane indicated above. **(b)** Gold standard Fourier shell correlation (GSFSC) curve after FSC-ask auto-tightening. **(c)** Orientation distribution plot (top) and posterior position directional distribution plot (bottom). **(d)** Histogram and directional FSC plot. Histogram (blue) shows the percentage of per angle FSC at the given spatial frequency. Global FSC (red) and ± 1 S.D. from mean directional FSC (green). Sphericity equals 0.928 out of 1. **(e)** Angular resolution map, and corresponding directional FSC volume (grey), in three perpendicular orientations. The relative angular resolution is indicated by the color bar.

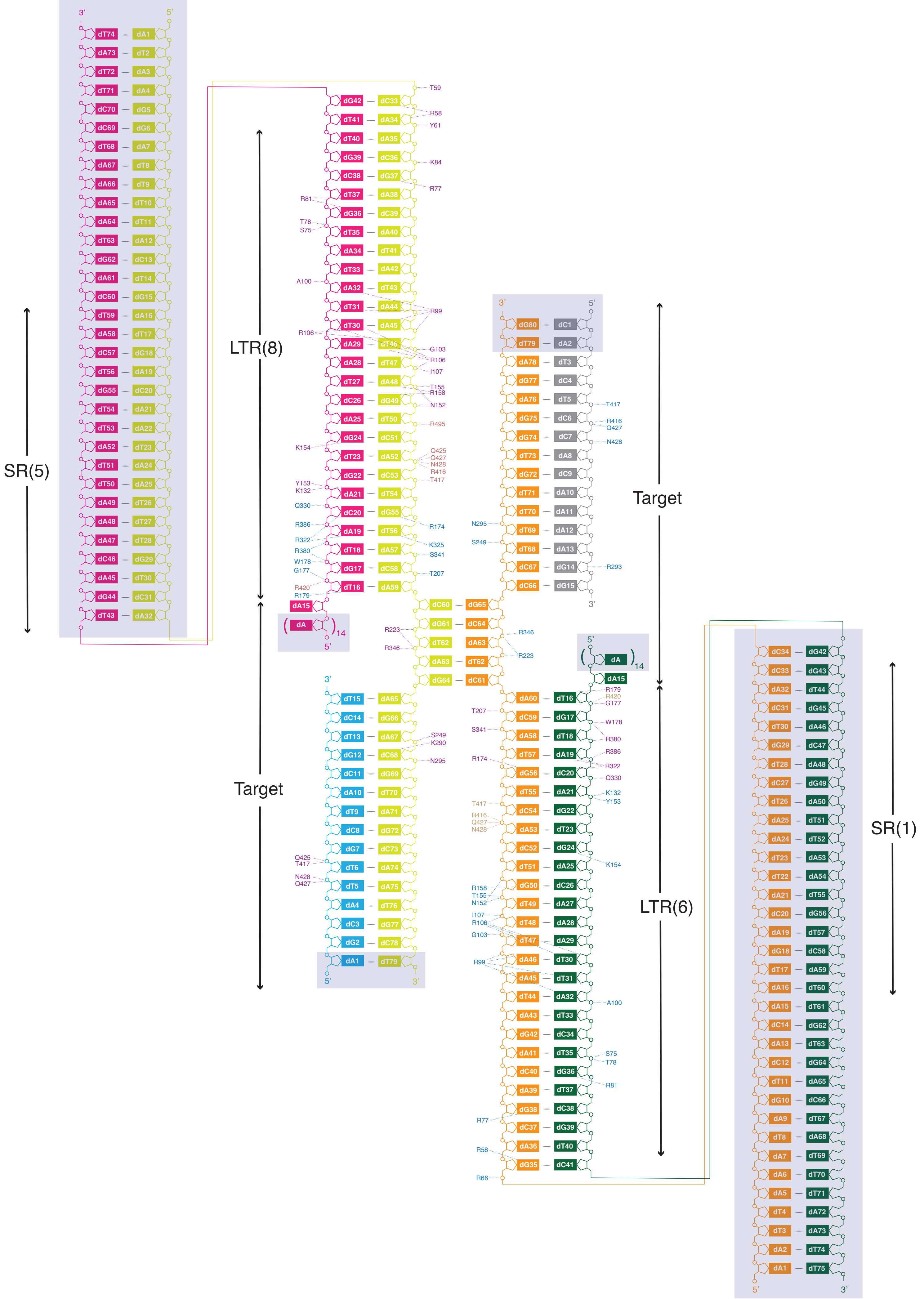

SR(5)
LTR(8)
Target
Target
LTR(6)
SR(1)

Supplementary Figure 7.- Protein-nucleic acids interactions in the shTnsB-STC complex. Polar contacts of the nucleic acids with the protein side and main chain are indicated. The colors of each chain correspond to the same colours used in Fig. 1c-1e and Supplementary Table 1. Lines between nucleotide bases indicate pairing. Note that the central nucleotides in the attachment site (dT62 and dA63) are not paired. Given that the attachment site contains 5 base pairs (an odd number), the DNA distortion caused by STC formation forces the central base pair to melt. The regions inside the blue squares are not visible in the cryo-EM map.

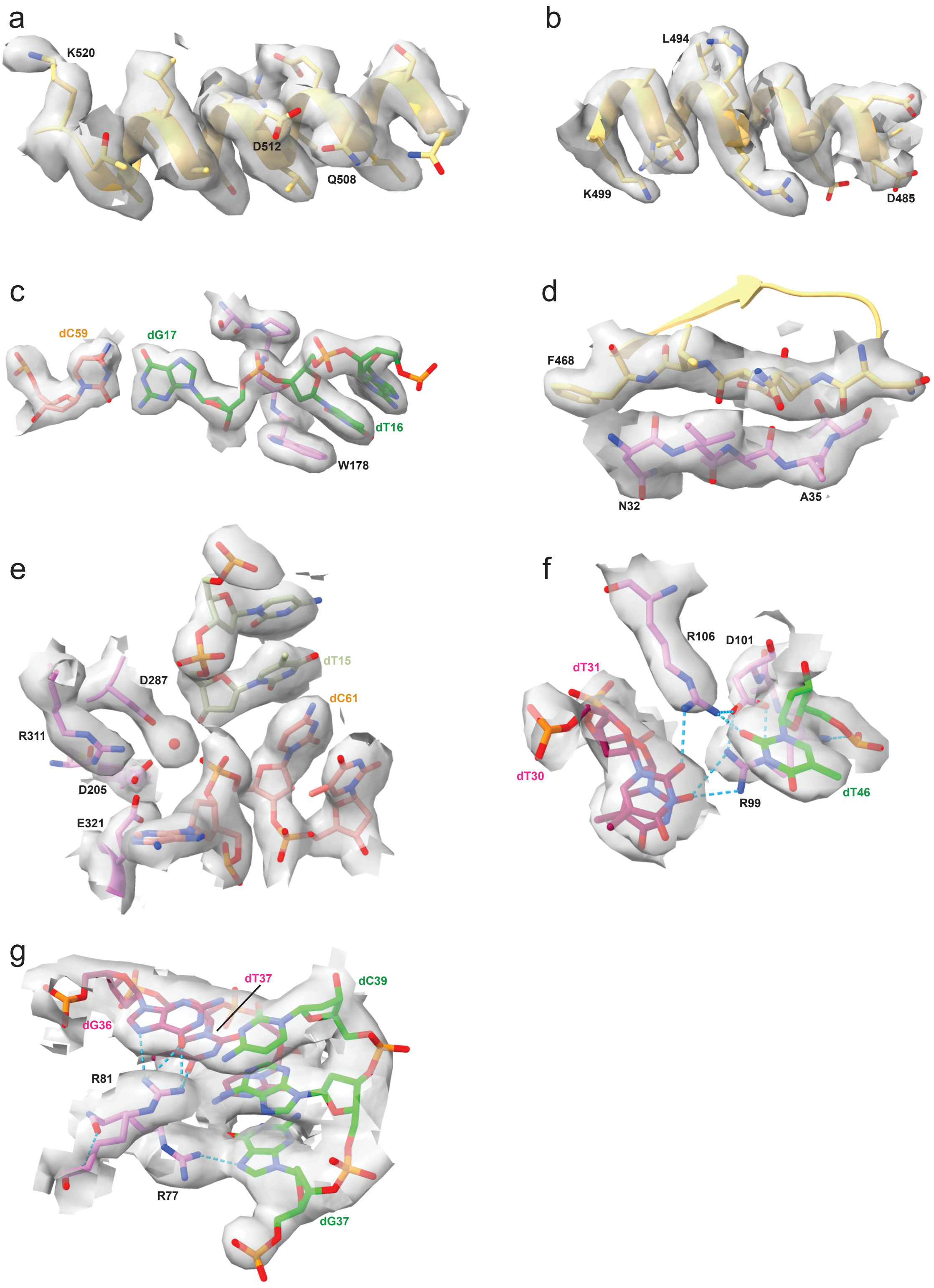

Supplementary Figure 8.- Fit of ShTnsB-STC atomic model to the cryo-EM map. Map regions are displayed at a 2 Å range within the displayed protein residues and nucleotides, at contour level 0.04 for protein-only panels, and level 0.1 for protein/DNA panels. Key residues and nucleotides are labelled. **(a)** OD α2, residues 504-520. **(b)** OD α1, residues 484-499. **(c)** Stacking interaction between W178, from the NTD2-DDM loop region, and dT16, from the overhand of the LE_polyA strand. **(d)** β-strands interaction, residues 32-36 and 464-468 of two neighboring chains, connecting the N-terminus with the MD domain of respective chains. **(e)** The DDE catalytic pocket and DNA, including the putative water molecule. **(f)** Polar interactions

of R99 and R106 from the NTD1-NTD2 linker region with DNA. **(g)** Polar interactions of the NTD1 domain R77 and R81 with DNA.

Supplementary Movie Legends

Supplementary Movie 1.- Conformational variability of the shTnsB-STC complex. The 3D variability analysis of the structure using cryoSPARC, shows that the core of the protein-DNA complex is quite stable while the arms of the X shaped DNA are highly flexible.

Supplementary Tables

Supplementary Table 1. Oligonucleotides used in this study. The bold nucleotides in the DNAs used to reconstitute the shTnsB-STC complex for cryo-EM denote the sequences visualized in the structure (see also Supp. Fig. 7)

Oligonucleotide	ssDNA or dsDNA	Sequence	Experiment	Synthesis
RE_Target – Chain I TS	ssDNA	ATAAGGATTTTACTGATGACAATAATTTGT<br>CA**CAACGACATATAATTAGTCACTGTACAC**<br>**GTAGAGACGTAGCAATGC**T	Cryo-EM	IDT
RE_polyA – Chain J NTS	ssDNA	AAAAAAAAAAAAAA**ATGTACAGTGACTAAT**<br>**TATATGTCGTTG**TGACAAATTATTGTCATC<br>AGTAAAATCCTTAT	Cryo-EM	IDT
Target_1 – Chain K	ssDNA	A**GCATTGCTACGTCT**	Cryo-EM	IDT
LE_Target – Chain L TS	ssDNA	AATTAAATAGTCACAATGACATTAATCTGT<br>CACC**GACGACAGATAATTTGTCACTGTACA**<br>**CTACGCCTTTTGTGGAGA**TG	Cryo-EM	IDT
LE_polyA – Chain M NTS	ssDNA	AAAAAAAAAAAAAA**ATGTACAGTGACAAAT**<br>**TATCTGTCGTC**GGTGACAGATTAATGTCAT<br>TGTGACTATTTAATT	Cryo-EM	IDT
Target_2 – Chain N	ssDNA	CA**TCTCCACAAAAGG**	Cryo-EM	IDT
LTR(6)-SR(1)	dsDNA	AATAGTCACAATGACATTAATCTGTCA<br>CCGACGACAGATAATTTGTCACTGTACACT<br>ACGCCTTT	EMSA	IDT
LE	dsDNA	ATTATATTGATGACATTTAATTTGTCATCA<br>ATTAATTAAGCAACGCTGATGGGTCACGAC<br>GACAATTAAATAGTCACAATGACATTAATC<br>TGTCACCGACGACAGATAATTTGTCACTGT<br>ACACTACGCCTTTTGTGG	EMSA	PCR
RE	dsDNA	TGCTACGTCTCTACGTGTACAGTGACTAAT<br>TATATGTCGTTGTGACAAATTATTGTCATC<br>AGTAAAATCCTTATACAGTATAGATTATAG<br>CGCTTTGGCAGTTTTAGCATAACCTCTTTG<br>CAGTGACAAAATAGATGTCGTTGTCCGTGA<br>TTGTGACAAATTAGCTGTCGCTTTGCAAGA<br>TAGGAAAAAGCTTTTGTGTATTTTCATAAT<br>GACAAATTGACTGTCGCAGGAGGTAA	EMSA	PCR
Donor	dsDNA	GTAAAACGACGGCCAGTGAATTGACGCGTA<br>TTGGGATGGAACAGCATTGCTACGTCTCTA<br>CGTGTACAGTGACTAATTATATGTCGTTGT<br>GACAAATTATTGTCATCAGTAAAATCCTTA<br>TACAGTATAGATTATAGCGCTTTGGCAGTT<br>TTAGCATAACCTCTTTGCAGTGACAAAATA<br>GATGTCGTTGTCCGTGATTGTGACAAATTA<br>GCTGTCGCTTTGCAAGATAGGAAAAAGCTT<br>TTGTGTATTTTCATAATGACAAATTGACTG<br>TCGCAGGAGGTAAATAATGGAGAAAAAAAT<br>CACTGGATATACCACCGTTGATATATCCCA<br>ATGGCATCGTAAAGAACATTTTGAGGCATT<br>TCAGTCAGTTGCTCAATGTACCTATAACCA<br>GACCGTTCAGCTGGATATTACGGCCTTTTT<br>AAAGACCGTAAAGAAAAATAAGCACAAGTT<br>TTATCCGGCCTTTATTCACATTCTTGCCCG<br>CCTGATGAATGCTCATCCGGAGTTCCGTAT<br>GGCAATGAAAGACGGTGAGCTGGTGATATG<br>GGATAGTGTTCACCCTTGTTACACCGTTTT<br>CCATGAGCAAACTGAAACGTTTTCATCGCT<br>CTGGAGTGAATACCACGACGATTTCCGGCA	EMSA	PCR

		GTTTCTACACATATATTCGCAAGATGTGGC<br>GTGTTACGGTGAAAACCTGGCCTATTTCCC<br>TAAAGGGTTTATTGAGAATATGTTTTTCGT<br>CTCAGCCAATCCCTGGGTGAGTTTCACCAG<br>TTTTGATTTAAACGTGGCCAATATGGACAA<br>CTTCTTCGCCCCCGTTTTCACTATGGGCAA<br>ATATTATACGCAAGGCGACAAGGTGCTGAT<br>GCCGCTGGCGATTCAGGTTCATCATGCCGT<br>TTGTGATGGCTTCCATGTCGGCAGAATGCT<br>TAATGAATTACAACAGTACTGCGATGAGTG<br>GCAGGGCGGGGCGTAATTTTTTTAAGGCAG<br>TTATTGGTGCCCTTCTAGAGTTTCAGTAAC<br>TATTAAACTTAGGGGTGGGTTGAAAGCAAG<br>TCCTTTTATCCGCTTGTTTTAATTGCTTTG<br>TATAATAATTGCAGAGCATATTATATTGAT<br>GACATTTAATTTGTCATCAATTAATTAAGC<br>AACGCTGATGGGTCACGACGACAATTAAAT<br>AGTCACAATGACATTAATCTGTCACCGACG<br>ACAGATAATTTGTCACTGTACACTACGCCT<br>TTTGTGGAGATGTCTAAGTTCCATCCCAAT<br>GGCGCGCCGAGCTTGGCGTAATCATGGTCA<br>TAGCTGTTTCCTG		

Supplementary Table2. Cryo-EM processing and model refinement

	TnsB-STC
EMD-15294	EMDB:
PDB 8AA5	PDB:
**Data collection**	
Electron Microscope	Titan Krios G2
Voltage (kV)	300
Electron detector	Falcon III
Electron dose (e-/Å^2)	40 (40 frms)
Nominal defocus range (μm)	-0.8 to -2.0
Pixel size (Å)	0.832
**3D Reconstruction**	
Raw images	4728
Initial particles	9646 K
Final particles	260 K
Map resolution (Å), 0.143 FSC	2.46 Å
Model resolution (Å), 0.5 FSC	2.8
Map sharpening B-factor (Å^2)	-74.5
Map sharpening method	DeepEMhancer
3DFSC sphericity	0.928
Local resolution range (Å)	~1.8 - 10
**Model composition**	
Protein residues	1486
Nucleotides	172
Non-hydrogen atoms	15509
Water	2
**Model refinement**	
ADP (Å^2)	
Protein	51.64
Nucleotide	47.93
Water	25.59
R.m.s. deviations	
Bond length (Å)	0.004
Bond angles (°)	0.580
Ramachandran statistics (%)	
Favored	95.91
Allowed	4.09
Outlier	0.00
Rotamer outlier (%)	4.62
MolProbity score	2.32
Clash score	12.42
Model vs Data CC (mask)	0.76
