# Supplementary material for: Structure of the TnsB transposase-DNA complex of type V-K CRISPR-associated transposon
Source: Nat Commun. 2022 Oct 2;13:5792. doi: 10.1038/s41467-022-33504-5 (PMC9527255; doi:10.1038/s41467-022-33504-5)
Supplement: Supplementary file 3 — Description of Additional Supplementary Files [file 41467_2022_33504_MOESM3_ESM.pdf]

Description of Additional Supplementary Files

File name: Supplementary Movie 1

Description: Conformational variability of the shTnsB-STC complex. The 3D variability analysis of the structure using cryoSPARC, shows that the core of the protein-DNA complex is quite stable while the arms of the X shaped DNA are highly flexible.
