# Supplementary figures and images for: Structure of the TnsB transposase-DNA complex of type V-K CRISPR-associated transposon
Source: Nat Commun. 2022 Oct 2;13:5792. doi: 10.1038/s41467-022-33504-5 (PMC9527255; doi:10.1038/s41467-022-33504-5)

**a**

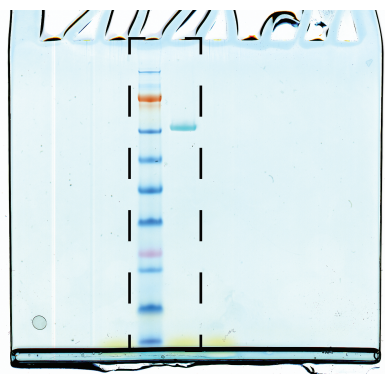

**c**

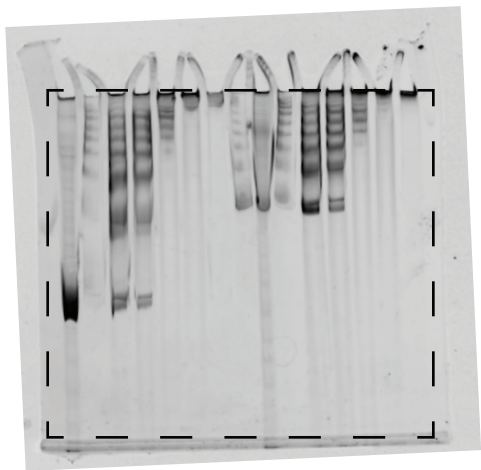

**d**

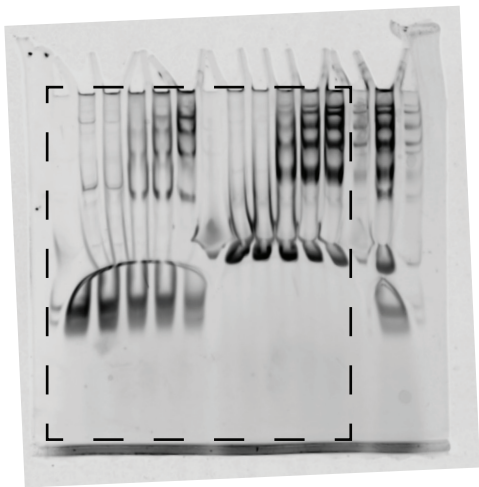

**e**

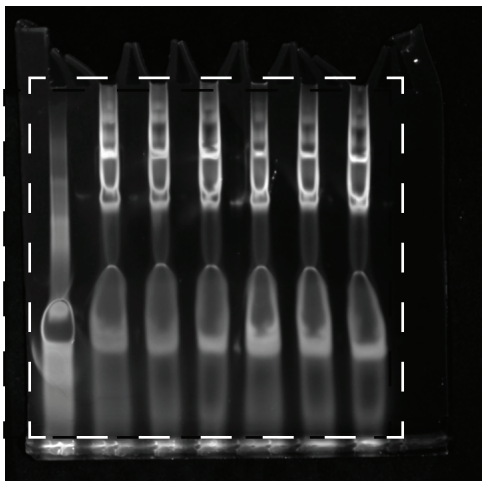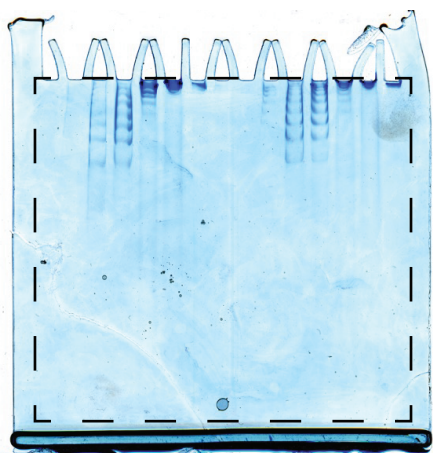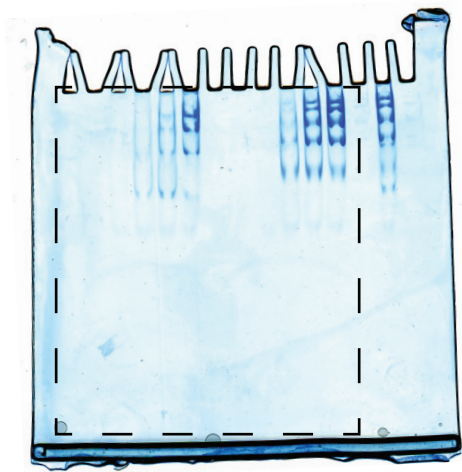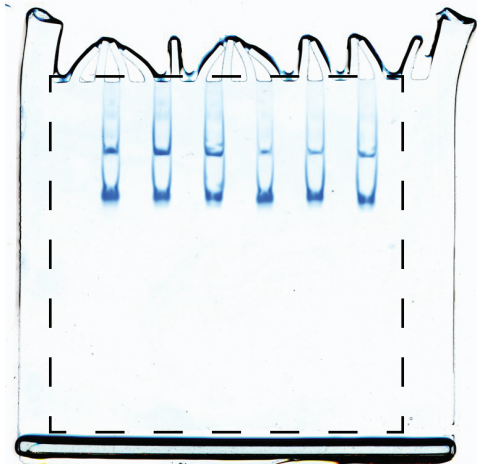

Supplement: Supplementary file 6 — Source Data [file 41467_2022_33504_MOESM6_ESM.zip › Source data-gels.pdf]
